# Deep learning for automated contouring of neurovascular structures on magnetic resonance imaging for prostate cancer patients

**DOI:** 10.1016/j.phro.2023.100453

**Published:** 2023-06-01

**Authors:** Ingeborg van den Berg, Mark H.F. Savenije, Frederik R. Teunissen, Sandrine M.G. van de Pol, Marnix J.A. Rasing, Harm H.E. van Melick, Wyger M. Brink, Johannes C.J. de Boer, Cornelis A.T. van den Berg, Jochem R.N. van der Voort van Zyp

**Affiliations:** a Department of Radiation Oncology, Division of Imaging & Oncology, University Medical Center Utrecht, Utrecht, The Netherlands; b Department of Urology, St. Antonius Hospital, Nieuwegein, Utrecht, The Netherlands; c Magnetic Detection and Imaging Group, Technical Medical Centre, University of Twente, Enschede, the Netherlands

**Keywords:** Artificial intelligence (AI), Contouring, Deep learning (DL), Magnetic resonance-guided radiotherapy (MRgRT), Neurovascular-sparing, Prostate cancer (PCa)

## Abstract

**Background and purpose:**

Manual contouring of neurovascular structures on prostate magnetic resonance imaging (MRI) is labor-intensive and prone to considerable interrater disagreement. Our aim is to contour neurovascular structures automatically on prostate MRI by deep learning (DL) to improve workflow and interrater agreement.

**Materials and methods:**

Segmentation of neurovascular structures was performed on pre-treatment 3.0 T MRI data of 131 prostate cancer patients (training [n = 105] and testing [n = 26]). The neurovascular structures include the penile bulb (PB), corpora cavernosa (CCs), internal pudendal arteries (IPAs), and neurovascular bundles (NVBs). Two DL networks, nnU-Net and DeepMedic, were trained for auto-contouring on prostate MRI and evaluated using volumetric Dice similarity coefficient (DSC), mean surface distances (MSD), Hausdorff distances, and surface DSC. Three radiation oncologists evaluated the DL-generated contours and performed corrections when necessary. Interrater agreement was assessed and the time required for manual correction was recorded.

**Results:**

nnU-Net achieved a median DSC of 0.92 (IQR: 0.90–0.93) for the PB, 0.90 (IQR: 0.86–0.92) for the CCs, 0.79 (IQR: 0.77–0.83) for the IPAs, and 0.77 (IQR: 0.72–0.81) for the NVBs, which outperformed DeepMedic for each structure (p < 0.03). nnU-Net showed a median MSD of 0.24 mm for the IPAs and 0.71 mm for the NVBs. The median interrater DSC ranged from 0.93 to 1.00, with the majority of cases (68.9%) requiring manual correction times under two minutes.

**Conclusions:**

DL enables reliable auto-contouring of neurovascular structures on pre-treatment MRI data, easing the clinical workflow in neurovascular-sparing MR-guided radiotherapy.

## 1 Introduction

Magnetic resonance-guided radiotherapy (MRgRT) is more frequently used as a treatment option for patients with localized prostate cancer (PCa) [1]. MRgRT provides soft tissue visualization and can account for inter and intra-fraction changes of the target volumes and organs-at-risk (OAR) [2]. In MR-guided adaptive PCa radiotherapy, daily recontouring of the target and surrounding tissue is essential for an optimal balance between the maximization of the target dose and the preservation of OAR [3].

Conventional OAR such as the bladder and rectum are delineated during the radiotherapy planning process to minimize the radiation dose in these organs and reduce the risk of acute and late genitourinary and gastrointestinal toxicities. Another common adverse effect is erectile dysfunction, which affects 25% to 55% of previously sexually functioning patients within 60 months after treatment [4]. Although sparing neural structures to improve erectile outcome is common practice in prostate surgery for more than two decades, this has only been introduced in recent years for image-guided radiotherapy [5], [6] to reduce the increased risk of late-onset erectile dysfunction associated with radiotherapy compared to normal age-related decline [7]. The regions of interest in neurovascular-sparing radiotherapy include the penile bulb (PB), the corpora cavernosa (CCs), the internal pudendal arteries (IPAs), and the neurovascular bundles (NVBs).

Various studies have assessed the agreement in manual contouring for neurovascular structures on prostate or pelvic MRI scans. Roach et al. [8] reported an average Dice similarity coefficient (DSC) of 0.66 for the PB and respectively 0.16 and 0.15 for the left and right NVB, while Cassidy et al. [9] reported an average DSC of 0.72 for the NVBs. Teunissen et al. [10] found a good interrater agreement for the inferior part of the NVBs (DSC = 0.67) but the median overall interrater DSC were respectively 0.60 and 0.61 for the left and right NVB and 0.59 for the IPAs.

These studies show that neurovascular structures may be difficult to contour and can result in considerable interrater disagreement. In addition, manual contouring of small elongated neurovascular structures is a labor-intensive process. Previous studies have established that deep learning (DL)-based delineation of conventional OAR can speed up the treatment planning procedure in prostate radiotherapy [11], [12]. However, no DL model has specifically focused on the segmentation of the neurovascular structures on prostate MRI for MRgRT. Therefore, we aimed to develop and validate DL models for automated contouring of neurovascular structures on pre-treatment prostate MRI as a first step for its application during online adaptive radiotherapy. Finally, a clinical evaluation of the use of DL-generated contours was performed among three radiation oncologists.

## 2 Materials and methods

### 2.1 Imaging data

We acquired pre-treatment 3.0 Tesla (T) T2-weighted MRI data of PCa patients undergoing MRgRT from our regional prospective registry (NCT04228211) between September 2020 and May 2022 at the University Medical Center Utrecht, The Netherlands. The study population comprised 147 patients with localized PCa who were treated with five fractions of 7.25 Gray on a 1.5 T MR-Linac system. We excluded seven patients due to metal artifacts and nine patients because of obliteration of the prostate-NVB interface at the rectoprostatic angle. A total of 131 PCa patients with a mean age of 68 (range: 62–75) years were included in this study, with most patients classified as intermediate-risk (n = 101), followed by low-risk (n = 17), and high-risk PCa (n = 13) according to the European Association of Urology (EAU) risk group classification. Pre-treatment MRI was performed on a 3.0 T Ingenia MR-RT system (Philips Healthcare, The Netherlands) with a three-dimensional (3D) turbo-spin echo sequence (repetition time = 1700 ms, echo time = 270 ms, flip angle = 50°, slice thickness = 2 mm, field of view = 400 × 446 × 180, reconstructed resolution = 0.62 × 0.62 × 2 mm, total acquisition time = 4 min 54 s). The MRI scans were randomly divided into a training cohort (80%) and a testing cohort (20%).

### 2.2 Contouring

An in-house developed contouring software package Volumetool was used for contouring. The delineated structures were the prostate, gross tumor volume (GTV), clinical target volume (CTV), planning target volume (PTV), bladder, rectum, seminal vesicles, femur (left and right), and pelvic bones. These structures were contoured by radiation oncologists on axial slices on the pre-treatment 3.0 T MRI and considered ground truth contours. The CTV was defined as the prostate, the base of the seminal vesicles, and the GTV with a 4 mm margin. For the PTV, a 5 mm isotropic margin was taken from CTV to PTV. The seminal vesicles were not routinely contoured, but in this study, they were completely contoured to enhance differentiation with the NVBs. The neurovascular structures, including PB, CC (left and right), IPA (left and right), and the NVB (left and right), were manually contoured by a single annotator (IB), after reaching consensus on the IPA and NVB contours for 25 patients of the training cohort with three radiation oncologists (JVZ, SP, MR), following the contouring atlas of the ERECT trial (Supplementary Material A) [6]. The IPAs and NVBs were contoured within the same craniocaudal extent as the PTV where accurate delineation is essential for optimal dose coverage. In total, we included 14 structures (i.e., prostate, bladder, rectum, seminal vesicles, left and right femur, pelvic bones, PB, left and right CC, left and right IPA, and left and right NVB), and these structures were labeled into voxel-based masks for model development.

### 2.3 Model development

Two 3D convolutional neural network (CNN) architectures, specifically nnU-Net and DeepMedic, were selected to perform auto-segmentation. We chose nnU-Net as state-of-the-art network architecture that automatically comprises the entire pipeline from pre-processing to post-processing without manual intervention and has been utilized successfully in many anatomical sites on MRI [13]. A 3D full resolution nnU-Net model was trained for 1000 epochs using a patch size of 32 × 224 × 224 voxels and a batch size of 2, with a Stochastic Gradient Descent optimizer and learning rate of 0.01. The model incorporated three pooling operations along the z-axis and five pooling operations along the x- and y-axes, and convolutional kernel sizes of [1 × 3 × 3, 3 × 3 × 3, 3 × 3 × 3, 3 × 3 × 3, 3 × 3 × 3, 3 × 3 × 3].

The second CNN utilized in this study was DeepMedic, which was initially developed for the segmentation of brain lesions on MRI scans [14] and was successfully implemented in our institution for MRI-based OAR auto-segmentation in prostate radiotherapy [11]. The DeepMedic network required pre-processing, which involved intensity normalization and resampling of voxel size to 1 × 1 × 1 mm. The network architecture comprised of four parallel spatial resolution pathways, including a primary pathway with an original resolution of 37^3^ voxels and three additional subsampled pathways, which were subsampled by factors of 3, 5, and 7 respectively. Each of the pathways consisted of 11 layers. The network was trained using the original configuration parameters, which included a batch size of 10, learning rate of 0.001, RMSprop optimizer with 0.6 momentum, 35 epochs, and L1 and L2 regularization with weights of 0.000001 and 0.0001, respectively. Volumetric DSC and Cross Entropy loss functions were used with identical weight factors. A one-voxel closing and hole filling operation was performed, followed by the selection of the largest 3D connected component to improve segmentation results. The DeepMedic segmentations were then resampled to the original voxel size for comparison with nnU-Net. Both networks were trained on a graphical processing unit (GPU) Quadro RTX 6000 (NVIDIA Corporation, USA).

### 2.4 Model evaluation

Segmentation performance was evaluated on the testing cohort in terms of volumetric DSC, mean surface distance (MSD), 95% Hausdorff distance (HD95), and surface DSC (SDSC) with 1 mm tolerance. Volumetric DSC is a measure of the spatial overlap between two segmentations, MSD for the average distances between two segmentations, HD95 for the maximum surface distances (95th percentile) between two segmentations, and SDSC for the surface agreement above a clinically determined tolerance parameter [15]. The quantitative metrics of the NVBs were evaluated within the same craniocaudal extent of the PTV, the prostate, and the inferior half (i.e., prostate midgland to the inferior border of the PTV) where the NVB is in closest proximity to the CTV. Surface distance metrics were calculated using the DeepMind Python package (https://github.com/deepmind/surface-distance). Wilcoxon-signed rank tests were conducted between the metrics of the two DL networks and significance was set at p ≤ 0.05. Non-normally distributed data were presented as median with interquartile range (IQR).

### 2.5 Clinical evaluation

Three prostate radiation oncologists (15, 10, 4 years of experience respectively) independently evaluated the DL-generated contours in 15 randomly selected patients of the testing cohort. All raters were asked to review the contours of the neurovascular structures and perform manual correction when necessary, once with the nnU-Net results and once with the DeepMedic results, for a total of 30 MRI scans per rater. All raters had access to the contouring atlas of the ERECT trial (Supplementary Material A) [6] and were blinded to the contours of the other raters. Interrater agreement was assessed for all rater pairs (i.e., three raters result in three rater pairs per patient) in terms of volumetric DSC, MSD, and HD95. Additionally, the manual correction time was recorded for all neurovascular structures collectively.

## 3 Results

### 3.1 Model performance

**Fig. 1:**
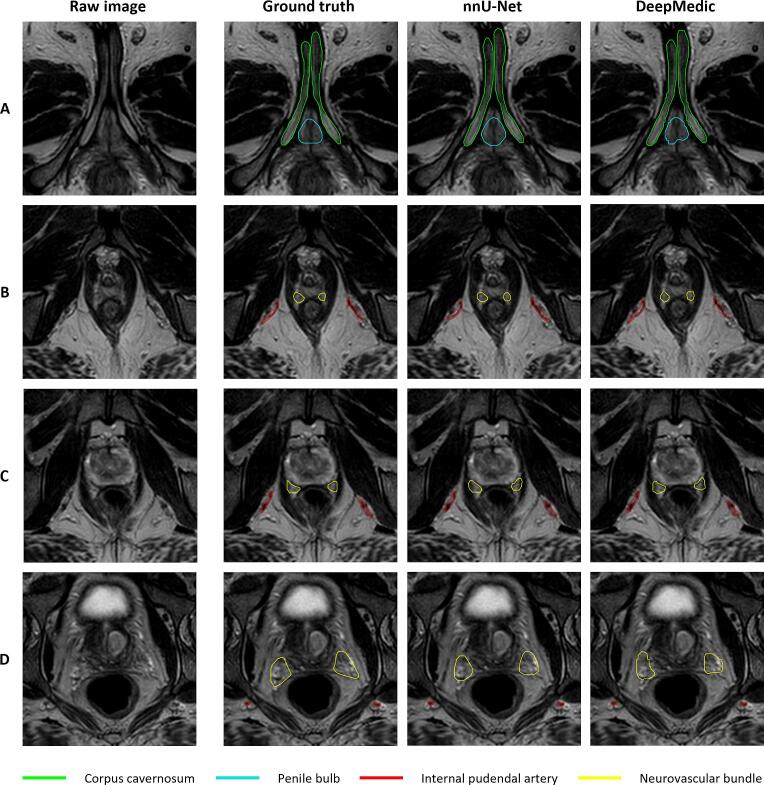
Representative case with the penile bulb (cyan), corpus cavernosum (green), internal pudendal artery (red), and neurovascular bundle (yellow) contours on T2-weighted MRI. Axial MR images obtained at the level of the penile bulb and corpora cavernosa (A), prostate apex level (B), prostate midgland level (C), and prostate base level (D). (For interpretation of the references to colour in this figure legend, the reader is referred to the web version of this article.)

The segmentation performances of nnU-Net and DeepMedic for the neurovascular structures in the testing cohort (n = 26) are listed in Table 1, and the DL-generated contours for a single patient of the testing cohort are presented in Fig. 1. nnU-Net achieved significantly higher median DSC values than DeepMedic for all neurovascular structures (p < 0.03), and demonstrated significantly higher median SDSC values for the PB (p = 0.02) and the NVBs at PTV and prostate level (p < 0.001).

**Table 1:** Quantitative evaluation of neurovascular structures for nnU-Net and DeepMedic segmentations.

	DSC		MSD		HD95		SDSC	
Structure	nnU-Net	DeepMedic	nnU-Net [mm]	DeepMedic [mm]	nnU-Net [mm]	DeepMedic [mm]	nnU-Net	DeepMedic
Penile bulb	0.92 (0.90–0.93)	0.91 (0.88–0.93)	0.27 (0.21–0.38)	0.33 (0.23–0.47)	1.93(1.34–2.21)	2.00(1.34–2.46)	0.90 (0.87–0.94)	0.88 (0.82–0.93)
Corpus cavernosum	0.90 (0.86–0.92)	0.88(0.86–0.91)	0.25 (0.20–0.36)	0.28 (0.23–0.37)	2.00(1.38–2.38)	2.00(1.40–2.76)	0.92 (0.87–0.95)	0.91 (0.88–0.94)
Internal pudendal artery	0.79(0.77–0.83)	0.72 (0.67–0.74)	0.24(0.17–0.29)	0.27(0.24–0.37)	2.00(1.74–2.45)	4.09(2.66–11.80)	0.95 (0.92–0.97)	0.95 (0.91–0.96)
Neurovascular bundle (PTV level)	0.77(0.72–0.81)	0.69(0.63–0.76)	0.71 (0.56–0.92)	0.89(0.77–1.12)	3.31(2.63–4.39)	4.38(3.23–7.77)	0.72(0.64–0.78)	0.65 (0.58–0.71)
Neurovascular bundle (prostate level)	0.80(0.75–0.84)	0.76 (0.68–0.80)	0.54 (0.42–0.72)	0.63(0.51–0.79)	2.50(1.99–3.37)	3.07(2.25–4.39)	0.79(0.70–0.85)	0.75 (0.68–0.80)
Neurovascular bundle (inferior half)	0.76 (0.71–0.82)	0.75 (0.68–0.79)	0.36 (0.29–0.53)	0.42(0.31–0.55)	1.99(1.40–2.74)	2.25(1.87–3.14)	0.87 (0.80–0.92)	0.85 (0.77–0.91)

Abbreviations: PTV = planning target volume; DSC = dice similarity coefficient; MSD = mean surface distance; HD95 = 95% boundary Hausdorff distance; SDSC = surface distance similarity coefficient with 1 mm tolerance. For the corpus cavernosum, internal pudendal artery, and neurovascular bundle: left and right are combined. Data are expressed as median (interquartile range).

The highest performance of both networks was achieved for the PB and CCs with median DSC values between 0.88 and 0.92 and median HD95 values between 1.93 and 2.00 mm. For the IPAs, nnU-Net achieved significantly higher median DSC values than DeepMedic (nnU-Net: 0.79 [IQR: 0.77–0.83], DeepMedic: 0.72 [IQR: 0.67–0.74], p < 0.001), but both networks demonstrated comparable performance in terms of SDSC (nnU-Net: 0.95 [IQR: 0.92–0.97], DeepMedic: 0.95 [IQR: 0.91–0.96]) when holding a tolerance level of 1 mm. The lowest performance was observed for the NVBs within the craniocaudal extent of the PTV, with median DSC values of 0.77 (IQR: 0.72–0.81) for nnU-Net and 0.69 (IQR: 0.63–0.76) for DeepMedic (p < 0.001). Both networks demonstrated higher DSC values when considering the NVBs at prostate level (nnU-Net: 0.80 [IQR: 0.75–0.84], DeepMedic: 0.76 [IQR: 0.68–0.80].

### 3.2 Clinical performance

**Fig. 2:**
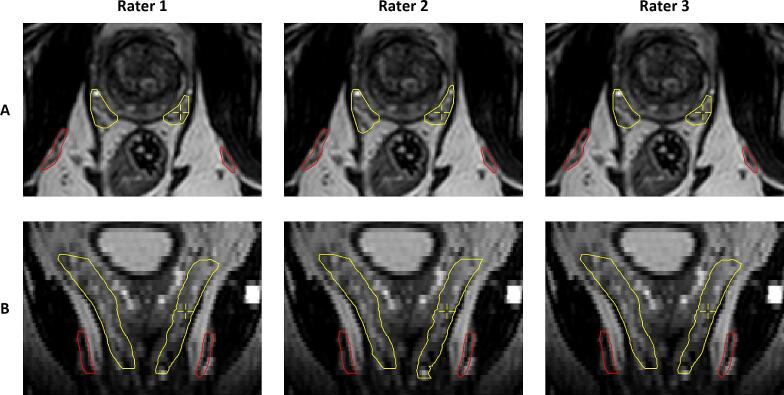
A representative example of the nnU-Net contour results after manual evaluation and correction for the internal pudendal arteries (red) and the neurovascular bundles (yellow) by three raters in the axial (A) and coronal (B) direction. Perfect interrater agreement was observed for the internal pudendal arteries between all raters and for the neurovascular bundles with rater 1 and 3. Rater 2 showed a Dice similarity coefficient of 0.90 for the left neurovascular bundle and 0.93 for the right neurovascular bundle with rater 1 and 3. (For interpretation of the references to color in this figure legend, the reader is referred to the web version of this article.)

**Fig. 3:**
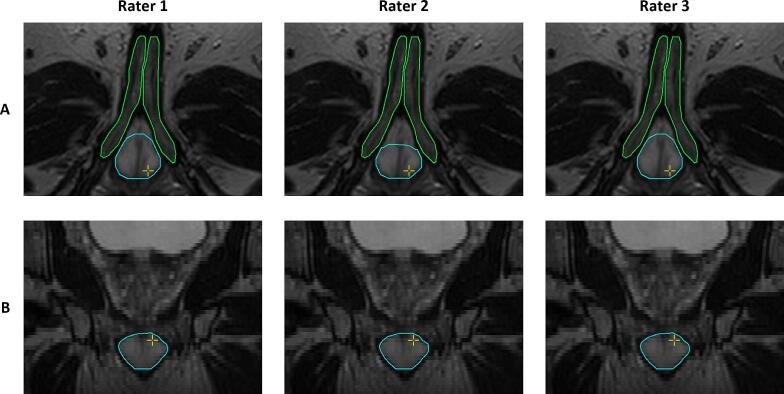
A representative example of the nnU-Net contour results after manual evaluation and correction for the penile bulb (cyan) and the corpora cavernosa (green) by three raters in the axial (A) and coronal (B) direction. Perfect interrater agreement was observed for the corpora cavernosa between all raters and for the penile bulb between rater 1 and 3. Rater 2 showed a Dice similarity coefficient of 0.93 for the penile bulb with rater 1 and 3. (For interpretation of the references to color in this figure legend, the reader is referred to the web version of this article.)

The interrater agreement among three radiation oncologists using DL-generated contours from nnU-Net and DeepMedic is shown in Table 2, Figs. 2, and 3. nnU-Net outperformed DeepMedic in terms of interrater DSC values for the PB, CCs, IPAs, and the NVBs at PTV and prostate level (p < 0.02). nnU-Net demonstrated median interrater DSC values between 0.93 and 1.00 and median interrater MSD between 0.00 and 0.26 mm across all neurovascular structures.

**Table 2:** Interrater agreement outcomes of the deep learning-generated contours of the neurovascular structures.

	DSC		MSD		HD95	
Structure	nnU-Net	DeepMedic	nnU-Net [mm]	DeepMedic [mm]	nnU-Net [mm]	DeepMedic [mm]
Penile bulb	0.93 (0.86–1.00)	0.87(0.82–1.00)	0.26 (0.00–0.46)	0.41(0.00–0.47)	2.35(0.00–4.43)	4.00 (0.00–6.03)
Corpus cavernosum	1.00 (1.00–1.00)	1.00 (0.98–1.00)	0.00 (0.00–0.00)	0.00 (0.00–0.00)	0.00 (0.00–0.00)	0.00 (0.00–0.02)
Internal pudendal artery	1.00(0.97–1.00)	0.93(0.85–0.98)	0.00(0.00–0.01)	0.04(0.01–0.13)	0.00 (0.00–0.62)	1.53 (0.15–2.72)
Neurovascular bundle (PTV level)	0.95(0.93–1.00)	0.88(0.82–0.97)	0.09 (0.01–0.16)	0.24(0.07–0.36)	1.40(0.00–1.88)	2.50 (0.68–3.86)
Neurovascular bundle (prostate level)	0.95(0.93–1.00)	0.95(0.89–0.99)	0.07 (0.01–0.11)	0.09(0.02–0.20)	1.25(0.00–1.76)	1.18 (0.00–2.33)
Neurovascular bundle (inferior half)	0.94 (0.91–1.00)	0.96 (0.89–1.00)	0.05 (0.00–0.09)	0.03(0.00–0.13)	1.18 (0.00–1.76)	0.62 (0.00–1.84)

Abbreviations: PTV = planning target volume; DSC = dice similarity coefficient; MSD = mean surface distance; HD95 = 95% boundary Hausdorff distance. For the corpus cavernosum, internal pudendal artery, and neurovascular bundle: left and right are combined. Data are expressed as median (interquartile range).

**Fig. 4:**
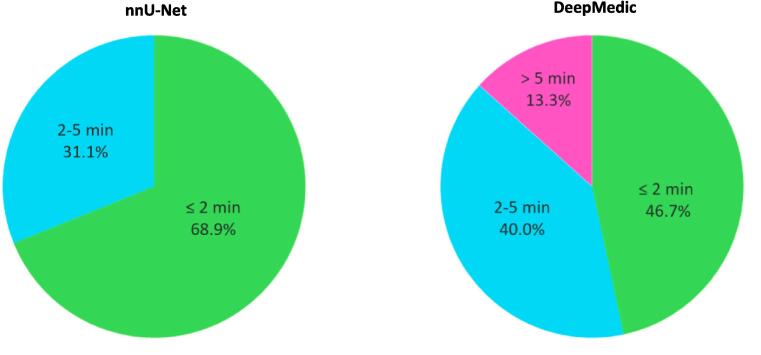
Pie chart with manual correction times for each DL-generated model of three radiation oncologists (3 × 15 patients = 45).

The interference time was approximately 51 s for nnU-Net and approximately 1 min and 23 s for DeepMedic. The total additional manual correction time for the nnU-Net contours was in 31/45 (68.9%) less than or equal to two minutes and in 14/45 (31.1%) between two and five minutes (Fig. 4). The total correction time for the DeepMedic contours was in 21/45 (46.7%) less than or equal to two minutes, followed by 18/45 (40.0%) between two and five minutes, and 6/45 (13.3%) for more than five minutes.

## 4 Discussion

To our knowledge, this study represents the first evaluation of AI to guide the preservation of neurovascular structures on 3.0 T prostate MRI for MRgRT. The DL-generated segmentations obtained with nnU-Net represented excellent overlap for the PB, CCs, IPAs, and the NVBs at prostate level with a median MSD below 0.54 mm and a median DSC ranging from 0.79 and 0.92. Optimal contouring of the neurovascular structures is essential to ensure that the neural and vascular tissues are sufficiently spared in unilateral or bilateral neurovascular-sparing MRgRT. The contouring performance for the NVBs at prostate level and the inferior half is clinically most relevant for neurovascular-sparing MRgRT because this part is in closest proximity to the CTV and lies within the high dose gradient region.

The NVBs are anatomically less concentrated and their shape becomes more divergent superior to the prostate, which led to a stronger deviation from the study ground truth contours and resulted in a median HD95 of 3.31 mm and DSC of 0.77. The contouring performance of the NVBs around the seminal vesicles may be considered clinically less relevant due to steep dose decay in the craniocaudal direction, which explains the higher DSC values in the clinical evaluation where radiation oncologists only revised DL-generated contours considered clinically relevant. The use of DL-generated contours with nnU-Net for the NVBs, IPAs, and CCs resulted in excellent interrater agreement among radiation oncologists, as demonstrated by a median interrater DSC of 0.95, 1.00, and 1.00, respectively. It indicates that the majority of cases did not require revision or resulted in the same revision. The variations were largest for the PB with a median HD95 of 2.35 mm, primarily in the ventral part (Fig. 3). This variability may be attributed to the lack of evaluations of the PB contours in the training cohort by multiple radiation oncologists. For neurovascular-sparing MRgRT, the superior part of the PB is clinically most relevant because this region may lie within the PTV. Consequently, variations in the ventral part may not impact the preservation of the PB but variations in the superior border should be as limited as possible.

The contouring agreement of the PB, IPAs, and NVBs on MRI data was also evaluated in previous studies. Roach et al. [8] obtained an average DSC of 0.66 for the PB and 0.15–0.16 for the NVBs, whereas Cassidy et al. [9] reported an average DSC of 0.72 for the NVBs on 3.0 T MRI data. Lack of specified training in contouring the PB and NVBs and differences in the extent contoured may have negatively affected interrater agreement. Furthermore, Teunissen et al. [10] evaluated the interrater agreement of the NVBs and IPAs of four raters on fifteen 1.5 T prostate MRI scans. Since the NVBs and IPAs are small elongated structures, the MSD may be a more representative parameter for the contouring overlap. The median MSD for the NVBs was around 1.9 mm for the NVBs, 1.1 mm for the inferior half of the NVBs, and 1.2 mm for the IPAs. Our clinical evaluation shows that auto-contouring leads to less interrater contouring variability than starting from scratch, with MSD values of 0.09–0.24 mm for the NVBs and 0.00–0.04 mm for the IPAs. Specifically, AI-assisted contouring leads to more consistency in the diameter of the contours in the axial plane compared to the previous study [10]. However, these improvements may not only be attributable to the use of AI but may also be associated with the higher signal-to-noise ratio available at 3.0 T compared to 1.5 T.

Furthermore, AI-assisted contouring is likely to be time-efficient for pre-treatment MRI because the total manual correction time was less than two minutes in 68.9% and 46.7% of the cases for nnU-Net and DeepMedic respectively. Although the time required for manual contouring of the neurovascular structures from scratch was not measured in our study, our clinical experience within the ERECT trial [6] typically shows a manual contouring time of approximately 10 min for accurate delineation of the neurovascular structures. These contours are propagated from 3.0 T to 1.5 T in neurovascular-sparing MRgRT using deformable image registration. Our clinical experience suggests that this method is feasible for visualizing the neurovascular structures on a lower field strength, with most of the propagated structures being well-registered. The DL-generated contours of nnU-Net show the potential application for online adaptive radiotherapy.

Automated contours of the NVBs may have potential applications beyond RT planning, such as the preoperative planning of PCa patients undergoing robot-assisted radical prostatectomy (RARP) [16]. The relation between the tumor and NVBs can be considered and used to establish a nerve-sparing indication. In addition, NVB segmentations can be incorporated into 3D prostate models, which already play an important role in surgical planning for RARP [17], [18]. Automated MRI-based NVB segmentations relieve the operator from performing labor-intensive manual segmentations and could be readily integrated into the clinical workflow of 3D MR-guided RARP.

There are some limitations associated with this study. First, only a subset of the ground truth contours in the training cohort was evaluated by multiple radiation oncologists but the insights gained from these evaluations were applied to the remaining cases. Second, we trained and evaluated the DL-generated contours on a 3.0 T MRI scanner. The DL-generated contours can already be propagated to 1.5 T but the use of auto-contouring is especially beneficial in the setting of online MRgRT on an MR-Linac, where contour adaptation in every fraction is known to improve treatment effectiveness and minimize dose to the NVBs [19]. Minimizing manual correction and interference time of DL networks is crucial for reducing possible intrafraction motion of the prostate during online adaptive radiotherapy. We aim to evaluate the DL network performance on conventional and neurovascular contours on a 1.5 T MR-Linac with quantitative, time-based (i.e., software generation time and manual correction time), and dosimetric metrics for an overall assessment of the clinical utility [15]. We could add uncertainty predictions to quickly flag DL-generated contours where manual inspection and possible corrections by radiation oncologists are needed [20], [21]. Finally, the model’s generalization ability should be evaluated by external validation, including institutes that define CTV/PTV margin criteria differently. The network performance and generalization ability can potentially be improved by including a larger training cohort that includes a wider variety of different neurovascular anatomies and contours of multiple radiation oncologists.

In conclusion, the use of DL for automatic delineations of neurovascular structures is feasible on prostate MRI data, easing the clinical workflow in neurovascular-sparing MRgRT. A good performance was reached for the PB, CCs, IPAs, and the NVBs at prostate level. Auto-contouring resulted in excellent interrater agreement on pre-treatment scans between radiation oncologists. Further research should evaluate the use of auto-contouring in online treatment planning during neurovascular-sparing MRgRT.

## Declaration of Competing Interest

The authors declare that they have no known competing financial interests or personal relationships that could have appeared to influence the work reported in this paper.
